# Automatic identification of relevant genes from low-dimensional embeddings of single-cell RNA-seq data

**DOI:** 10.1093/bioinformatics/btaa198

**Published:** 2020-03-24

**Authors:** Philipp Angerer, David S Fischer, Fabian J Theis, Antonio Scialdone, Carsten Marr

**Affiliations:** b1 Institute of Computational Biology, Helmholtz Zentrum München - German Research Center for Environmental Health, Neuherberg 85764, Germany; b2 TUM School of Life Sciences Weihenstephan, Technical University of Munich, Freising 85354, Germany; b3 Institute of Epigenetics and Stem Cells, Helmholtz Zentrum München - German Research Center for Environmental Health, Neuherberg 85764, Germany; b4 Institute of Functional Epigenetics, Helmholtz Zentrum München - German Research Center for Environmental Health, München 81377, Germany

## Abstract

**Motivation:**

Dimensionality reduction is a key step in the analysis of single-cell RNA-sequencing data. It produces a low-dimensional embedding for visualization and as a calculation base for downstream analysis. Nonlinear techniques are most suitable to handle the intrinsic complexity of large, heterogeneous single-cell data. However, with no linear relation between gene and embedding coordinate, there is no way to extract the identity of genes driving any cell’s position in the low-dimensional embedding, making it difficult to characterize the underlying biological processes.

**Results:**

In this article, we introduce the concepts of local and global gene relevance to compute an equivalent of principal component analysis loadings for non-linear low-dimensional embeddings. *Global gene relevance* identifies drivers of the overall embedding, while *local gene relevance* identifies those of a defined sub-region. We apply our method to single-cell RNA-seq datasets from different experimental protocols and to different low-dimensional embedding techniques. This shows our method’s versatility to identify key genes for a variety of biological processes.

**Availability and implementation:**

To ensure reproducibility and ease of use, our method is released as part of destiny 3.0, a popular R package for building diffusion maps from single-cell transcriptomic data. It is readily available through Bioconductor.

**Supplementary information:**

Supplementary data are available at *Bioinformatics* online.

## 1 Introduction

Single-cell RNA-sequencing (scRNA-seq) has massively improved the resolution developmental trajectories Baron *et al.* (2016) and allowed unprecedented insights into the heterogeneity of complex tissues Tritschler *et al.* (2017) and Vento-Tormo *et al.* (2018). On the flip side, new challenges have arisen due to the amount of data that needs to be processed Angerer *et al.* (2017), higher levels of technical and biological noise Yuan *et al.* (2017), and identification and interpretation of known and novel cell types Pliner *et al.* (2019). To exploit the new opportunities and deal with the new challenges, a large number of algorithms and tools have been developed Zappia *et al.* (2018).

Dimension reduction methods create a low-dimensional embedding of the high-dimensional gene expression space. Those embeddings are widely used for two applications: They (i) serve as a visual overview of the data on which gene expression profiles and per-cell or per-cluster statistics can be compared. They (ii) serve as inputs for further downstream computational analysis. For example, principal component analysis (PCA) is a popular technique to identify orthogonal linear combinations of genes that explain variance in the data. PCA loadings quantify the contribution of genes to each principal component and help understand the genetic drivers of the underlying molecular processes. However, linear methods are often not able to capture the complexity of high-dimensional datasets (Haghverdi *et al.*, 2015), which is why nonlinear dimension reduction methods have become the standard for scRNA-seq data analysis [see e.g. t-SNE (t-Stochastic Neighborhood Embedding) Husnain *et al.* (2019), diffusion maps; Coifman *et al.* (2005), Haghverdi *et al.* (2015) and Husnain *et al.* (2019); UMAP (Uniform Manifold Approximation and Projection) Becht *et al.* (2018) and McInnes *et al.* (2018); and graph-based methods Islam *et al.* (2011)]. However, no intrinsic measure of individual genes’ contribution to each embedding dimension exists for non-linear embeddings. Without such a measure, the identification of genes that drive the variability in the data requires tedious manual inspection and prior knowledge about possible target genes.

Here, we introduce *gene relevance*, a measure for a gene’s contribution to variance in low-dimensional embeddings, and present a method to infer a local as well as a global gene relevance score from any kind of low-dimensional embedding. To demonstrate the utility of the method, we apply gene relevance to several datasets prepared with different droplet- and plate-based protocols (see Supplementary Table S1). Gene relevance is available as part of the R package *destiny*Angerer *et al.* (2016).

## 2 Materials and methods

**Fig. 1.**
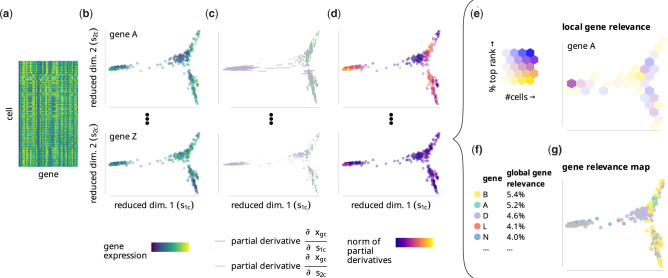
The gene relevance concept. (**a**) A gene expression matrix from a scRNA-seq experiment is (**b**) reduced to a low-dimensional embedding *s_pc_*, with each dot representing a cell, and the color representing the expression *x_gc_* of gene g∈{A,B,…,Z} in cell *c*. (**c**) Expression changes are calculated from estimates of partial derivatives with respect to the embedding, which results in one value per cell×gene×dimension combination. (**d**) We score the relevance of each gene in each cell according to the partial derivatives’ euclidean norm. This score indicates how relevant each gene is within its neighborhood. (**e**) For local gene relevance scores, we subdivide the embedding into bins and determine the fraction of cells per bin for which a given gene is among the (e.g. 10) most relevant genes (indicated by ‘%top rank’ in the figure legend). We color bins of the embedded cells according to their local gene relevance score, and fade them according to the number of cells they contain (indicated by ‘#cells’ in the legend). (**f**) For the global gene relevance score, we determine local relevance for all cells instead of a bin. In our illustrative example, Gene B has been ranked among the top 10 genes in 5.4% of all cells. (**g**) A gene relevance map indicates all cells where a given gene has the largest norm of partial derivatives (with or without a smoothing step—see Section 2). Such cells mark the areas where that gene has high local relevance

We define gene relevance as a measure of how much a gene contributes to the cell-to-cell variability in a low-dimensional embedding of a scRNA-seq dataset (see Fig. 1). It can be interpreted as a generalization of PCA loadings to non-linear dimensionality reduction techniques. Note that PCA loadings are constant with respect to the PC space while feature importance in a non-linear embedding is naturally a non-constant function of the embedding coordinates. A ranking of genes based on their relevance is built for every cell of the embedding. These rankings can be combined to obtain a measurement of the *local* or *global* relevance of each gene (see Fig. 1e–f), which highlight genes relevant in defined sub-regions of the embedding and all cells, respectively. To explore and visualize the results further, the method also provides a *gene relevance map*, where the locally most relevant genes are displayed along with their corresponding neighborhoods in the embedding (see Fig. 1g). Below, we describe in details every step in the estimation of global and local gene relevance.

### 2.1 Neighborhoods

If a k nearest neighbor (kNN) search has been performed as part of the embedding, it can be efficiently used for estimating the gene relevance. To perform the kNN search, destiny offers the choice between euclidean distance, cosine distance, and spearman rank correlation distance. The latter was used in all analyses performed for this article.

### 2.2 Gene expression changes

The differential *d_gc_* of gene *g* in cell *c* describes the change in gene expression *x_gc_* along a change in embedding coordinates *s_pc_*, where p∈{1,…,P} is the embedding dimension and *d_gc_* corresponds to the partial derivatives of the gene expression with respect to each embedding coordinate:

(1) $$d_{gc}=\left(\frac{\partial x_{gc}}{\partial s_{1c}},\ldots ,\frac{\partial x_{gc}}{\partial s_{Pc}}\right)$$

We estimated *d_gc_* from the cells’ neighborhood ${\text{NN}}_{k}(c)$ in gene expression space using finite differences. To address the high dropout rate present in scRNA-seq data, we do not define *d_gc_* for *x_gc_* = 0.

(2) $${\hat{\left(d_{gc}\right)}}_{p}=\left\{ \begin{array}{cc} NA, & \text{if}\;x_{gc}=0\\ \underset{n\in {\text{NN}}_{k}(c)\wedge n\neq c}{\text{median}}\frac{x_{gc}-x_{gn}}{s_{pn}-s_{pc}}, & \text{otherwise} \end{array}\right.$$

### 2.3 Local gene relevance

The basis of local and global gene relevance is the score of gene g∈{1,…,G} in cell c∈{1,…,C}, defined as the euclidean norm $||d_{gc}|{|}_{2}$ of the differential *d_gc_*:

(3) $$\left||d_{gc}\right|{|}_{2}=\sqrt{\sum_{p=1}^{P}{\left(d_{gc}\right)}_{p}^{2}}$$

In each cell *c*, genes can be ranked according to their score $||d_{gc}|{|}_{2}$, from most to least relevant. Given the ranks ${\text{rg}}_{||d_{gc}|{|}_{2}}$ of gene *g* and a rank cutoff ${\text{rg}}_{\max}$, we define the local gene relevance $LR_{{\text{rg}}_{\max}}(g,\Psi )$ of a gene for a set of cells Ψ⊆{1,…,C} as:

(4) $${\text{LR}}_{{\text{rg}}_{\max}}(g,\Psi )=\frac{\sum_{c\in \Psi }\left[{\text{rg}}_{||d_{gc}|{|}_{2}}<{\text{rg}}_{\max}\right]}{\left|\Psi \right|}$$

with the Iverson bracket notation

(5) $$\left[P\right]=\left\{\begin{array}{l} \begin{array}{cc} 1, & \text{if}\;P\;\text{is}\;\text{true} \end{array}\\ \begin{array}{cc} 0, & \text{otherwise} \end{array} \end{array}\right.,\;\text{for}\;\text{any}\;\text{statement}\;P$$

In our analyses, we used the default ${\text{rg}}_{\max}=10$. The method is robust against the cutoff used, with the most relevant genes stable even for higher cutoffs >100 (see Supplementary Fig. S7).

### 2.4 Global gene relevance

The global relevance ${\text{GR}}_{{\text{rg}}_{\max}}(g)$ can simply be defined as the local gene relevance for the set of all cells {1,…,C}.

(6) $${\text{GR}}_{{\text{rg}}_{\max}}(g)={\text{LR}}_{{\text{rg}}_{\max}}(g,\{1,\ldots ,C\})$$

### 2.5 Local gene relevance plots

**Fig. 2.**
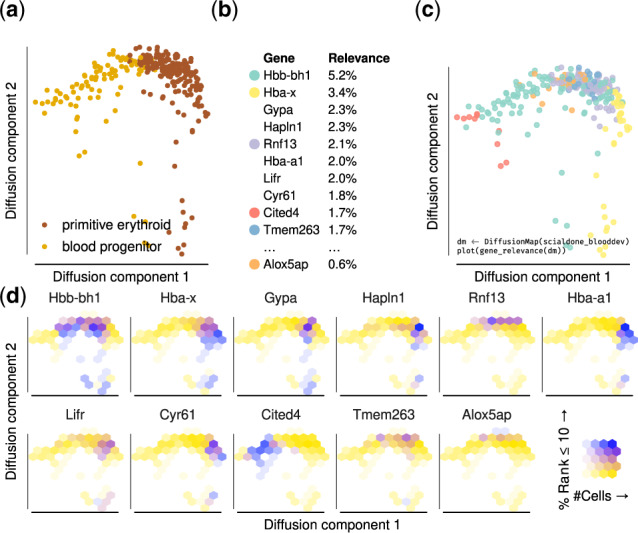
Gene relevance automatically detects drivers of embryonic blood development. (**a**) Diffusion map of 271 single hematopoietic progenitor cells from mostly Day 7.5 and 7.75 mouse embryos, profiled in Scialdone *et al.* (2016). (**b**) Global gene relevance identifies Hbb-bh1 and Hba-x as genes that change most dramatically during hematopoietic development. (**c**) A gene relevance map identifies the contribution of relevant genes in specific regions of the process and the corresponding code to create it. The genes corresponding to each color are shown in panel (b). (**d**) A local gene relevance plot details the areas where the contribution of genes is highest. Alox5ap shows a high local relevance in the top region of the diffusion map and has been implicated with early blood development Ibarra-Soria *et al.* (2018)

The local gene relevance of genes can be visualized by evenly dividing the embedding space into bins b∈{1,…,B} and calculating $LR_{{\text{rg}}_{\max}}(g,\Psi_{b})$ for the set of cells falling into each bin $\Psi_{b}$. This visualization is shown in Figures 1e and 2d.

### 2.6 Gene relevance maps

For a set of genes of interest Ω⊆{1,…,G} (which can be chosen, e.g. among those with highest global relevance) and each cell *c*, we define the locally most relevant gene $l_{c}^{m}$ after a number of smoothing steps *m*:

(7) $$l_{c}^{m}=\underset{g\in \Omega }{\text{arg}\;\text{max}}\left\{ \begin{array}{cc} ||d_{gc}|{|}_{2}, & \text{if}\;m=0\\ \frac{1}{k}\sum_{n\in NN_{k}(c)}\left[l_{n}^{m-1}=g\right], & \text{otherwise}. \end{array}\right.$$

During a smoothing iteration, we replace the local gene relevance score of cell *c* and gene *g* with the fraction of neighbors that have *g* as the most relevant gene.

The amount of smoothing is controlled by the smoothing parameter *m*. Decreasing *m* will result in more locality and more genes with a high relevance in a small region will appear on the map, while genes with a medium relevance in more or larger regions will vanish. When determining the locally most relevant gene using the globally most relevant genes as Ω, the aforementioned parameter ${\text{rg}}_{\max}$ can be used as a sensitivity parameter, with higher values resulting in more genes being selected as relevant. Finding the globally most relevant genes are robust to both parameters (see Supplementary Fig. S7), while locally relevant genes are affected to a larger extent. Importantly, due to the short computation time of gene relevance maps, one can explore several combinations of parameters. For cell sizes in the range of few hundreds, the running time is less than one to a few seconds with 40 000 cell×gene combinations per second processed. For cell sizes in the thousands 20 000 cell×gene combinations per second are processed, resulting in run times of a few minutes (run times have been measured on a single 3.6 GHz CPU core).

### 2.7 scRNA-seq data

We demonstrate our method on four datasets (see Section 3 and Supplementary Table S1 for details).

In the mouse gastrulation data from Scialdone *et al.* (2016), we used count data from 271 cells mostly of the neural plate (embryonic Day 7.5) and head fold (embryonic Day 7.75) development stages of mouse embryos. There, the libraries were constructed using the Smart-seq2 protocol, read counts were obtained via HTseq-count. The 271 cells we used correspond to the clusters annotated as ‘blood progenitor’ and ‘primitive erythroid’ in the original publication. We selected highly variable genes using the method of Brennecke *et al.* (2013) because of its stable performance Yip *et al.* (2018), and embedded the log-transformed data using the diffusion map implementation destiny Angerer *et al.* (2016).

For the two human cell datasets, we applied the same analysis steps, starting from the highly variable gene selection (see Supplementary Fig. S3). The human endocrine cell data from Veres *et al.* (2019) was sequenced using the inDrops platform, while the human brain organoid data from Gray Camp *et al.* (2015) used the SMARTer Ultra Low RNA Kit in combination with an Illumina sequencer. The data from Kolodziejczyk *et al.* (2015) used Nextera kits together with an Illumina sequencer. For further details about the pre-processing of these data, please refer to the individual publications.

## 3 Results

We demonstrate our method on a scRNA-seq dataset of blood progenitors and blood cells from mouse embryos Scialdone *et al.* (2016; see Fig. 2a). In the original publication, these data were used to reconstruct a trajectory representing primitive erythropoiesis, along which blood marker expression increases and other markers (such as endothelial cells) decrease. There, an ad hoc method was devised to find important genes in the 2D diffusion map embedding of the data. Here, we show how our method can be used ‘out of the box’ to rank genes based on their local and global relevance.

First, we ranked all highly variable genes according to their global gene relevance (see Fig. 2b). As expected, the high-ranking genes are mostly associated with blood development, including the hemoglobin genes Hba-a1, Hba-x, Hbb-bh1 and the erythrocyte membrane genes Gypa and Cited4 Yahata *et al.* (2002). The genes Cyr61 and Hapln1 are involved in extracellular matrix and important for development of the cardiovascular system Latinkić *et al.* (2001). The top of the list has a good overlap with the ad hoc method in Scialdone *et al.* (2016): 4 genes are shared between the top 10 of both lists, and we find a Rank-Biased Overlap of *RBO_p_* = 0.48, where we used *p *=* *0.9, which assigns ∼86% of the weight to the first 10 genes Webber *et al.* (2010).

Second, we created a gene relevance map (Fig. 2c). Five out of the six locally most relevant genes (see Fig. 2c) are among the ten most globally relevant ones. Interestingly, Alox5ap is included only in the gene relevance map, because its contribution is confined to a small region of the diffusion space (bottom right panel in Fig. 2d) and hard to detect at the level of gene expression (see Supplementary Fig. S1). This gene was not discovered by the ad hoc method of Scialdone *et al.* (2016), but it has been recently found to be important in early blood development Ibarra-Soria *et al.* (2018). Locally and globally relevant genes can also be inferred in other embeddings such as t-SNE van der Maaten and Hinton (2008) and UMAP Becht *et al.* (2018), with a stable recovery of the most relevant genes between comparable embeddings (see Supplementary Fig. S2).

Applied to other scRNA-seq datasets, we showcase versatility and ease of application of our method. In droplet-sequenced data of human endocrine cells Veres *et al.* (2019), gene relevance maps detect genes driving the separation of sub-regions of the embedding (see Supplementary Fig. S3a), in accordance to the markers identified in the original paper. In human brain organoid cells Gray Camp *et al.* (2015), we detect relevant genes different from the markers specified in the article because of a low-density region between mesenchymal cells and neurons/neural progenitors (see Supplemetnary Fig. S3b). The genes therefore seem to be selected for driving the difference between progenitors and neurons: TXNRD1 plays a vital role for neuron progenitor cells Soerensen *et al.* (2008), the selenoprotein SELT protects neurons against oxidative stress in mouse models Boukhzar *et al.* (2016), and CRABP1 modulates the neuronal cell cycle in mice Lin *et al.* (2017).

Finally, we applied gene relevance to mouse embryonic stem cells grown in three different pluripotency retaining media Kolodziejczyk *et al.* (2015). As expected for cells in a relatively homogenous pluripotent steady state, the relevant genes for diffusion map embedding of all three media were enriched for housekeeping, metabolic and proliferation pathways (see Supplementary Fig. S5).

## 4 Discussion

We presented a method that is able to reliably detect relevant genes from low-dimensional embeddings of scRNA-seq data. More specifically, our method computes both a local and a global gene relevance score: local gene relevance identifies the main drivers of the cell-to-cell variability in defined sub-regions of the embedding, while global gene relevance identifies those of the whole embedding. In addition to a gene ranking based on global relevance, the method also provides graphic tools to visualize the local gene relevance (see Fig. 1e) and the changes in gene expression levels within the embedding (see Fig. 1c and Supplementary Fig. S1). It can be used for any single-cell dataset and any dimensionality reduction technique.

We applied our method to three datasets, including one from mouse embryonic blood progenitors, where we show that it performs comparably to a technique custom-made for the dataset. Interestingly, our method identifies Alox5ap (Fig. 2), a gene that was recently shown to be important for blood development in a later publication Ibarra-Soria *et al.* (2018). In two other examples, we used human cells, endocrine Veres *et al.* (2019) and from brain organoids Gray Camp *et al.* (2015), showing that the method works robustly in varied conditions.

When compared with the classical method of looking at PCA loadings, gene relevance provides a generalization not tied to this dimension reduction method, as it is also suited for non-linear dimension reduction methods. It can therefore be used to explore the differences in the embeddings obtained by different dimension reduction methods (see Supplementary Fig. S2). Applied to a PCA embedding, gene relevance recovers a similar list of genes to those with the highest PC loadings (see Supplementary Fig. S8). Other methods to identify important genes specifically from scRNA-seq data exist, but most of them aim to find marker genes that can best distinguish different cell types Delaney *et al.* (2019). Conversely, the method we presented is unsupervised and does not rely on cell type annotation.

Recently, two computational methods have been developed to identify variable genes in spatial RNA-seq datasets, trendsceek and SpatialDE Edsgärd *et al.* (2018) and Svensson *et al.* (2018). Although these methods were designed to find patterns in spatial transcriptomic datasets, they can also be used to identify relevant genes in low-dimensional embeddings of scRNA-seq datasets [see Supplementary Fig. S6 in Edsgärd *et al.* (2018)]. We compared our approach to trendsceek and found similar genes (see Supplementary Fig. S6) in a considerably shorter running time: our method took 6.5 s, whereas trendsceek needed 1080 s (run times measured on a single 2.0 GHz CPU core). SpatialDE returned a perfect score for too many genes, making gene ranking impossible. This is probably related to both methods being optimized toward identifying spatial patterns. Moreover, neither method allows estimation of local gene relevance.

To summarize, our gene relevance method is a fast and versatile exploratory tool that can help identify the biological processes and reveal the presence and driving genes of potentially rare cell sub-populations. It is available online, easily applicable and faster than model fitting approaches. Although we focused our discussion on scRNA-seq datasets, our method can be applied to virtually any kind of dataset where low-dimensional embeddings are obtained, including, for instance, single-cell epigenomic Shema *et al.* (2019) and mass cytometry data Spitzer and Nolan (2016).

Gene relevance has been developed as part of the Bioconductor package *destiny*: bioconductor.org/packages/destiny. API documentation for analysis and plotting and are available at is at https://theislab.github.io/destiny/

The datasets analyzed within this publication are available from their original publications as described in Supplementary Table S1

## Supplementary Material

btaa198_Supplementary_Data
