# Supplementary material for: Automatic identification of relevant genes from low-dimensional embeddings of single-cell RNA-seq data
Source: Bioinformatics. 2020 Mar 24;36(15):4291–5. doi: 10.1093/bioinformatics/btaa198 (PMC7520047; doi:10.1093/bioinformatics/btaa198)
Supplement: btaa198_Supplementary_Data [file btaa198_supplementary_data.zip › btaa198-suppl_data/gene-relevance-supplementary-.pdf]

Bioinformatics
Advance Access Publication Date: 4th of September 2019
Supplementary Material

Automatic identification of relevant genes from low-dimensional embeddings of single cell RNAseq data

Supplementary Material

Angerer *et al.*

Supplementary figures and tables for the paper *Automatic identification of relevant genes from low-dimensional embeddings of single cell RNAseq data*

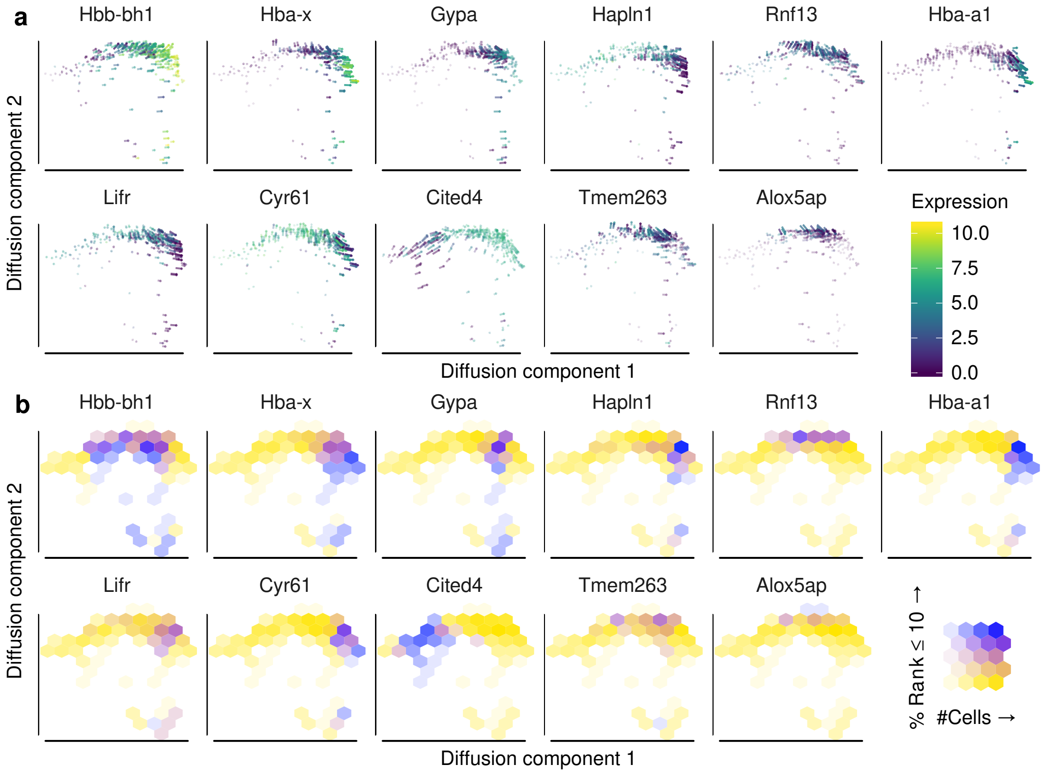

Suppl. Fig. 1. Gene relevance automatically identifies drivers of a developmental process. (a) Expression levels and corresponding differentials of selected genes and (b) local gene relevance maps for the same genes in a diffusion map of the embryonic blood dataset. Obvious gene expression changes can be identified from mapping the expression in single cells in a low dimensional embedding (e.g. for Cited4, Cyr61, Hba-a1), while the direction and strength (line length) of the differential simultaneously visualizes gene expression changes (a). Subtle, but also important gene expression changes are more robustly identified with our concept of gene relevance (e.g. Alox5ap, Tmem263, Rnf13) as explained in Fig. 1a (b).

© The Author 2019. Published by Oxford University Press. All rights reserved. For permissions, please e-mail: journals.permissions@oup.com

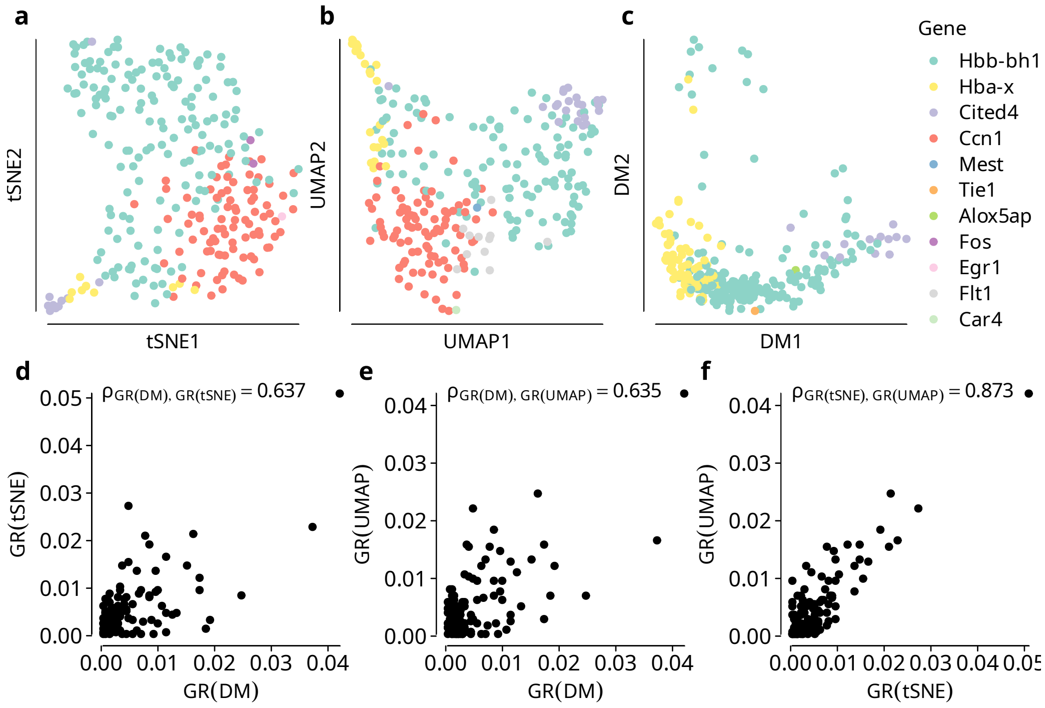

Suppl. Fig. 2. (a-c) Gene relevance maps of (a) t-SNE, (b) UMAP and (c) diffusion map embeddings of the embryonic blood dataset, with the top ranking locally relevant genes indicated on a gene relevance map. While the embeddings have differently sized regions driven by genes their gene relevance maps share, comparable lists of genes are identified, and some patterns are conserved (e.g., Hba-x and Cited4 marking opposite ends of both embeddings). Note that the gene relevance map of the diffusion map embedding differs from the one in Fig. 2c, as it was created from only the two displayed diffusion components to mirror the two-dimensional t-SNE and UMAP. (d-f) Correlation plots for global gene relevance scores in different embeddings. The similar UMAP and t-SNE have a high correlation between the scores (~0.87), while their correlation to the diffusion map's scores is medium-high (~0.64).

Publication	Description	Protocol	URL	Figures
Scialdone *et al.* (2016)	Mouse embryo gastrulation (271 cells used)	Smart-seq2	`http://gastrulation.stemcells.cam.ac.uk/scialdone2016`	Fig. 2, Suppl. Figures 1, 2, 4 and 6 to 8
Veres *et al.* (2019)	In vitro differentiating human β cell progenitors (4207 cells used)	inDrops	`https://github.com/meltonlab/scbeta_indrops`	Suppl. Fig. 3
Gray Camp *et al.* (2015)	Human cerebral organoids (412 cells used)	Illumina C1 + SMARTer	`https://www.ncbi.nlm.nih.gov/geo/download/?acc=GSE75140`	Suppl. Fig. 3
Kolodziejczyk *et al.* (2015)	Undifferentiated mouse ES cells (704 cells)	Illumina C1 + SMARTer	`https://espresso.teichlab.sanger.ac.uk/`	Suppl. Fig. 5

Suppl. Table 1. Analyzed data sets. In the Scialdone et al. (2016) data, we analyzed the blood progenitor a mesoderm subsets.

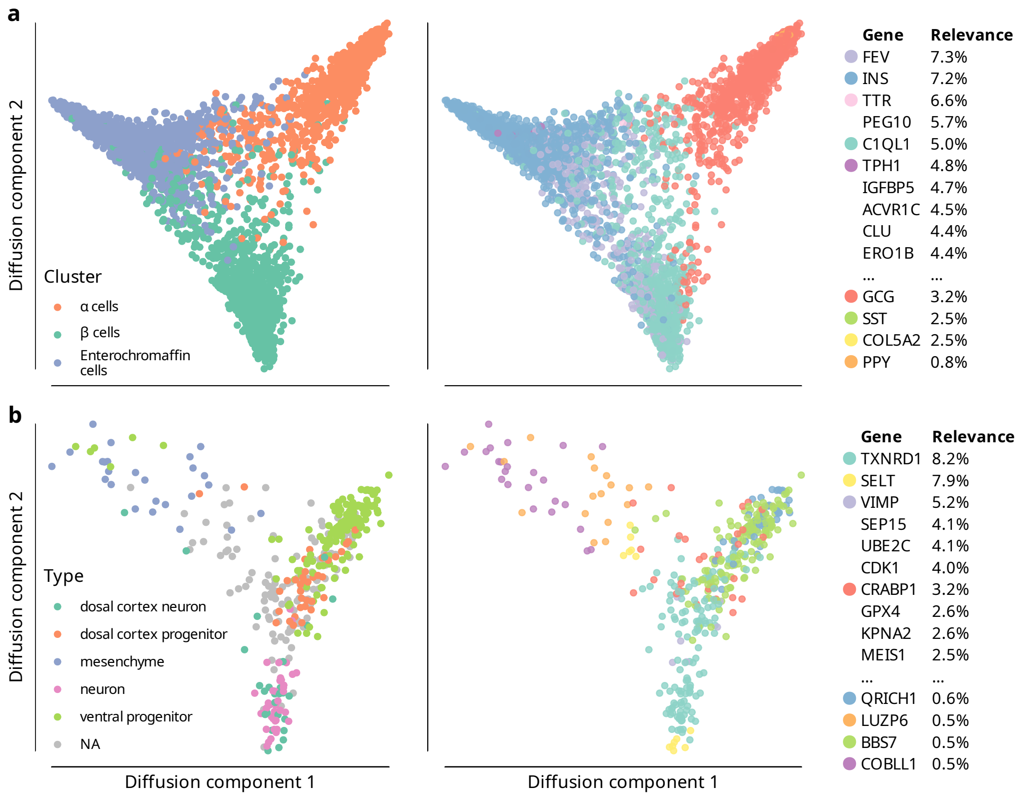

Suppl. Fig. 3. Gene relevance maps automatically identify drivers of tissue heterogeneity and differentiation in two human scRNAseq datasets. (a) Reaggregated stem cell derived endocrine cells Veres et al. (2019). FEV, GCG, INS, and TPH1 are mentioned as markers for the respective cell types in the original paper. DLK1 was manually excluded due to its dominating of the gene relevance map. The gene relevance map reveals that FEV mostly drives the distinction between enterochromaffin (EC) and β cells, while GCG takes a similar role for EC vs. α cells. (b) Embryonic stem cell derived brain organoids Gray Camp et al. (2015). The relevant genes TXNRD1, SELT, VIMP, and CRABP1 are known to play roles in neuronal proliferation and differentiation.

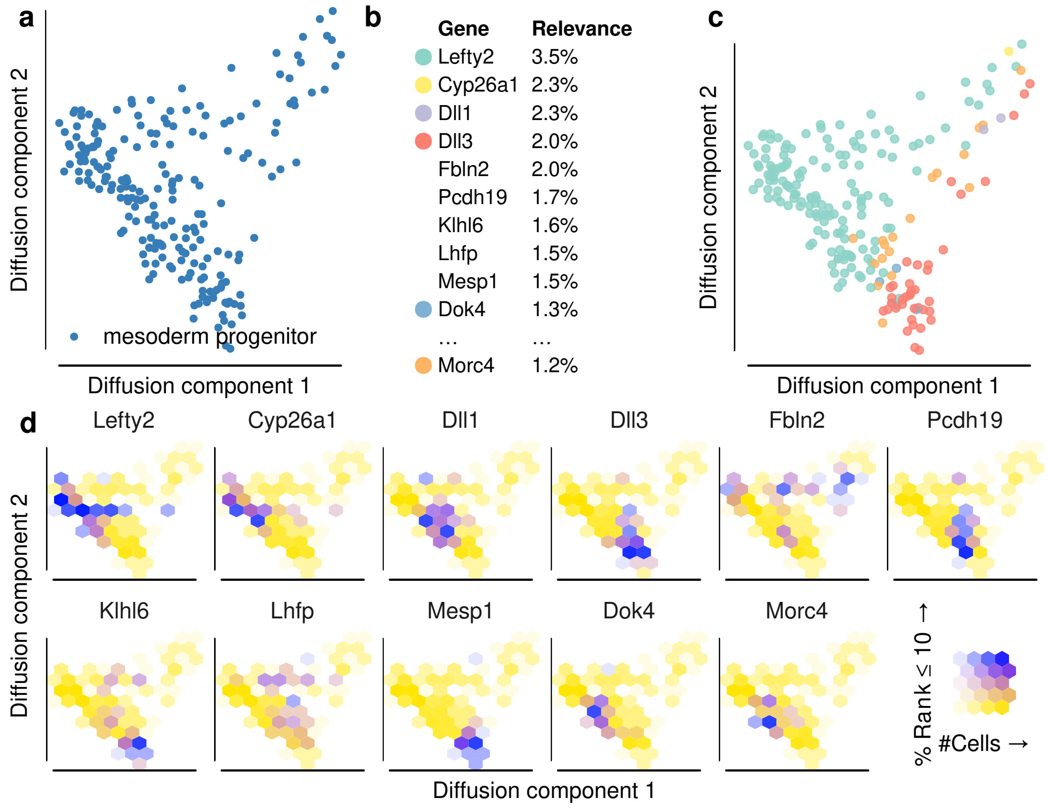

Suppl. Fig. 4. Gene relevance successfully identifies relevant genes in mesodermal progenitor cells. (a) Diffusion map of 216 mesodermal progenitor cells profiled in Scialdone et al. (2016). (b) List of globally most relevant genes in the diffusion map and (c) corresponding gene relevance map. (d) Local gene relevance plots for the most locally and globally relevant genes, showing relevance patterns not visible on the gene relevance map (c).

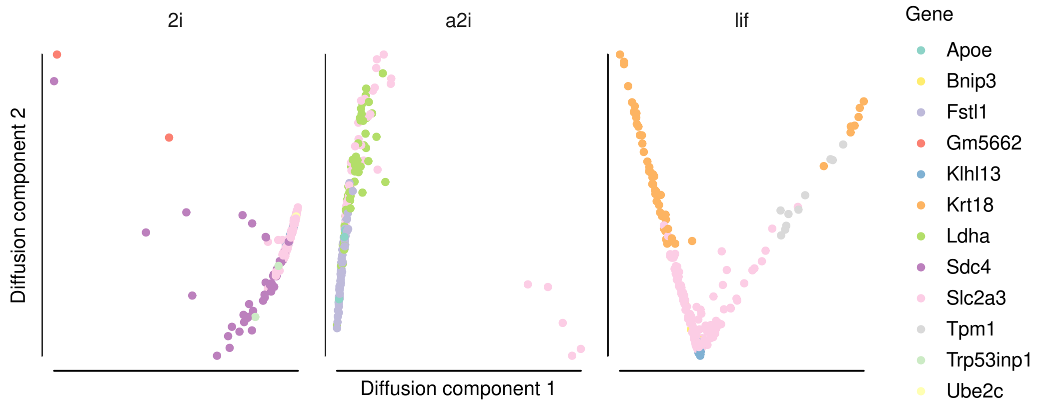

Suppl. Fig. 5. Gene relevance maps of mouse embryonic stem cells grown in 3 different pluripotency maintaining media (2i, a2i, and Lif, indicated on top of each plot). Data from Kolodziejczyk et al. (2015). As expected, the identified relevant genes are responsible for housekeeping and other regular cellular functions: The top 3 enriched gene ontology terms retrieved by GOrilla Eden et al. (2009) for the three media are: 2i: cell division, positive regulation of cellular protein catabolic process, negative regulation of mitotic cell cycle, a2i: response to toxic substance, lactate biosynthetic process from pyruvate, response to oxidative stress, lif: negative regulation of cellular process, negative regulation of biological process, cellular component organization.

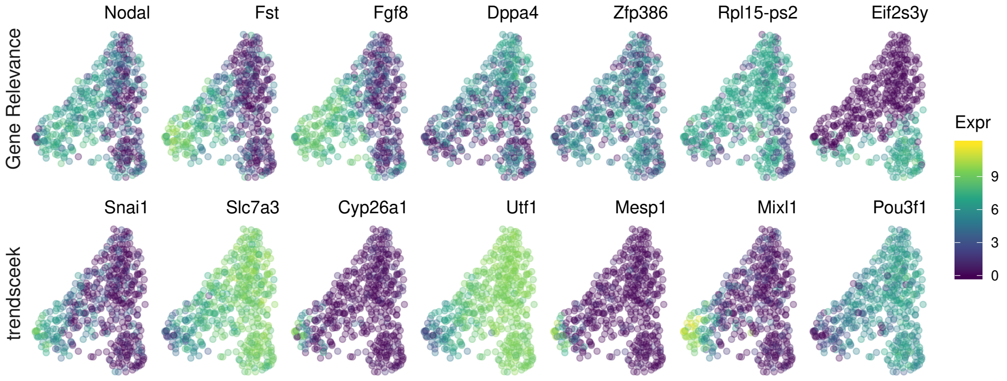

Suppl. Fig. 6. Trendsceek Edsgärd et al. (2018) and global gene relevance were applied to identify relevant genes from a UMAP embedding of epiblast cells from Scialdone et al. (2016). The figure shows the expression levels of the top relevant genes identified by global gene relevance (top row) and trendsceek (bottom row) overlaid to a t-SNE embedding similar to one from the trendsceek paper. Both lists include genes that play important roles in gastrulation (e.g., T, Mixl1, Fgf8, Frzb, etc), as expected for epiblast cells at this stage of development Scialdone et al. (2016).

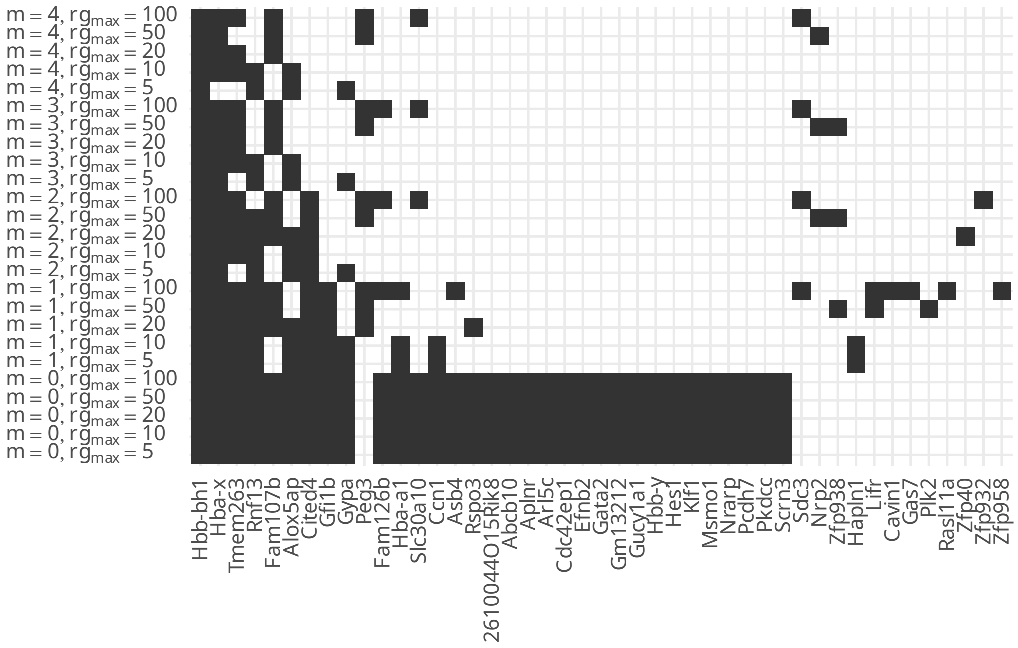

Suppl. Fig. 7. Gene relevance maps robustly display similar gene sets even when strongly modifying its parameters. Strongly correlated genes will often have no fully stable place in rankings when modifying parameters. We therefore recommend using gene relevance maps with interactive visualizations such as Plotly or Vega to be able to see the local relevance ranking for each cell in a gene relevance map or each bin in a local relevance plot. Nevertheless, gene relevance maps are surprisingly stable when modifying the two gene relevance specific parameters: Cutoff (rg_{max}) and smoothing (m). The exception is turning off smoothing completely ($m = 0$) and thereby preserving all initially found genes.

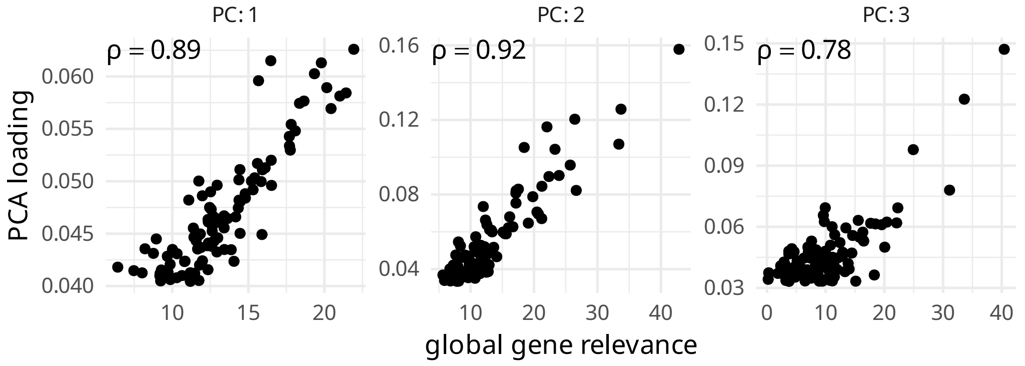

Suppl. Fig. 8. Gene relevance generalizes PCA loadings for the nonlinear case. Due to gene relevance using a local kernel defined by a kNN search, the resulting genes – while stongly correlated – are not equivalent.

References

Eden, E., Navon, R., Steinfeld, I., Lipson, D., and Yakhini, Z. (2009). GOrilla: a tool for discovery and visualization of enriched GO terms in ranked gene lists. *BMC Bioinformatics*, **10**, 48.

Edsgärd, D., Johnsson, P., and Sandberg, R. (2018). Identification of spatial expression trends in single-cell gene expression data. *Nat. Methods*, **15**(5), 339–342.

Gray Camp, J., Badsha, F., Florio, M., Kanton, S., Gerber, T., Wilsch-Bräuninger, M., Lewitus, E., Sykes, A., Hevers, W., Lancaster, M., Knoblich, J. A., Lachmann, R., Pääbo, S., Huttner, W. B., and Treutlein, B. (2015). Human cerebral organoids recapitulate gene expression programs of fetal neocortex development. *Proc. Natl. Acad. Sci. U. S. A.*, **112**(51), 15672–15677.

Kolodziejczyk, A. A., Kim, J. K., Tsang, J. C. H., Ilicic, T., Henriksson, J., Natarajan, K. N., Tuck, A. C., Gao, X., Bühler, M., Liu, P., Marioni, J. C., and Teichmann, S. A. (2015). Single cell RNA-Sequencing of pluripotent states unlocks modular transcriptional variation. *Cell Stem Cell*, **17**(4), 471–485.

Scialdone, A., Tanaka, Y., Jawaid, W., Moignard, V., Wilson, N. K., Macaulay, I. C., Marioni, J. C., and Göttgens, B. (2016). Resolving early mesoderm diversification through single-cell expression profiling. *Nature*, **535**(7611), 289–293.

Veres, A., Faust, A. L., Bushnell, H. L., Engquist, E. N., Kenty, J. H.-R., Harb, G., Poh, Y.-C., Sintov, E., Gürtler, M., Pagliuca, F. W., Peterson, Q. P., and Melton, D. A. (2019). Charting cellular identity during human in vitro β-cell differentiation. *Nature*, **569**(7756), 368–373.
