# Supplementary material for: Automatic identification of relevant genes from low-dimensional embeddings of single-cell RNA-seq data
Source: Bioinformatics. 2020 Mar 24;36(15):4291–5. doi: 10.1093/bioinformatics/btaa198 (PMC7520047; doi:10.1093/bioinformatics/btaa198)
Supplement: btaa198_Supplementary_Data [file btaa198_supplementary_data.zip › btaa198-suppl_data/supp-fig3.pdf]

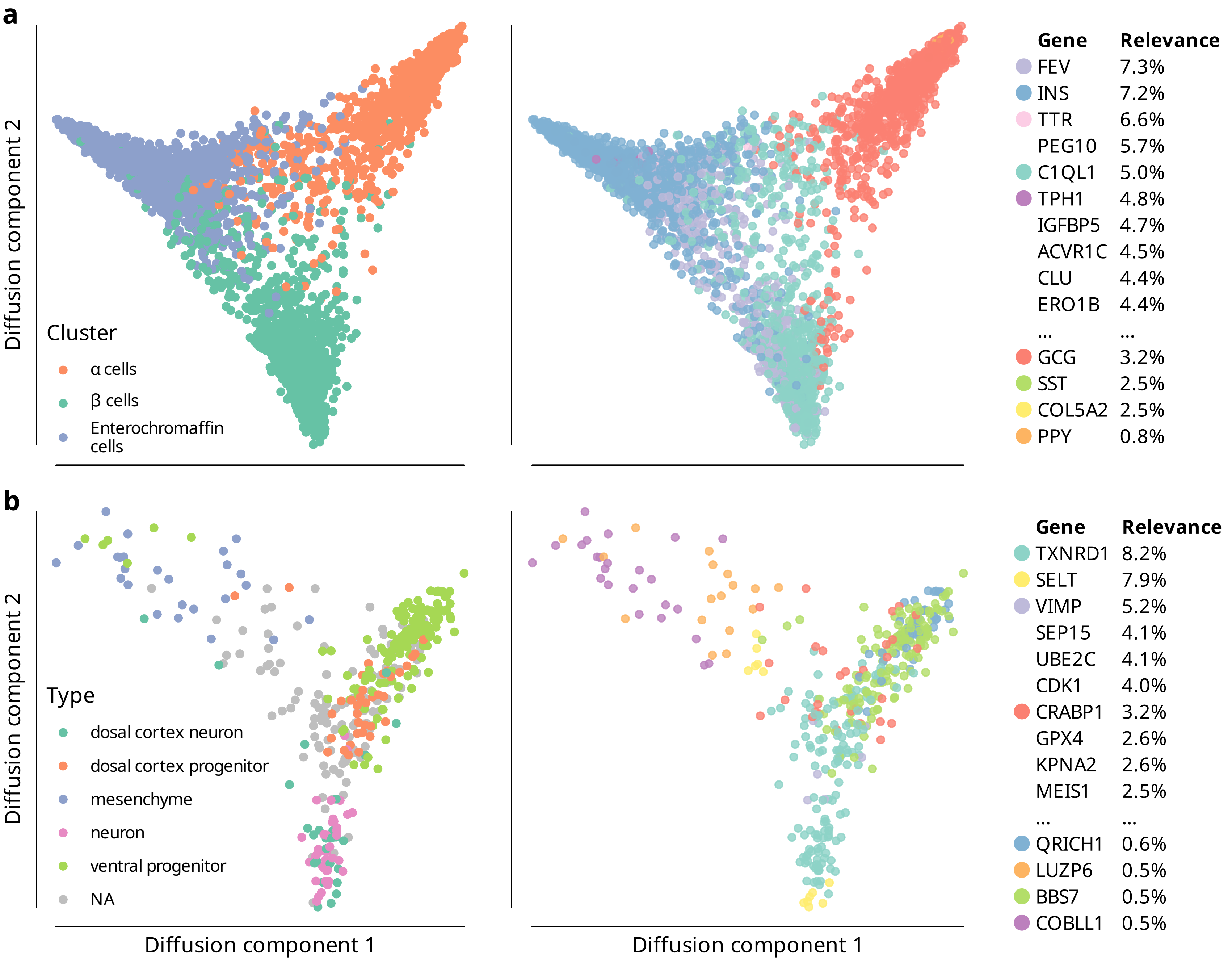
a
Diffusion component 2
Cluster
α cells
β cells
Enterochromaffin cells
Gene Relevance
FEV 7.3%
INS 7.2%
TTR 6.6%
PEG10 5.7%
C1QL1 5.0%
TPH1 4.8%
IGFBP5 4.7%
ACVR1C 4.5%
CLU 4.4%
ERO1B 4.4%
... ...
GCG 3.2%
SST 2.5%
COL5A2 2.5%
PPY 0.8%
b
Diffusion component 2
Type
dosal cortex neuron
dosal cortex progenitor
mesenchyme
neuron
ventral progenitor
NA
Diffusion component 1
Diffusion component 1
Gene Relevance
TXNRD1 8.2%
SELT 7.9%
VIMP 5.2%
SEP15 4.1%
UBE2C 4.1%
CDK1 4.0%
CRABP1 3.2%
GPX4 2.6%
KPNA2 2.6%
MEIS1 2.5%
... ...
QRICH1 0.6%
LUZP6 0.5%
BBS7 0.5%
COBLL1 0.5%
