# Supplementary material for: Automatic identification of relevant genes from low-dimensional embeddings of single-cell RNA-seq data
Source: Bioinformatics. 2020 Mar 24;36(15):4291–5. doi: 10.1093/bioinformatics/btaa198 (PMC7520047; doi:10.1093/bioinformatics/btaa198)
Supplement: btaa198_Supplementary_Data [file btaa198_supplementary_data.zip › btaa198-suppl_data/supp-fig5.pdf]

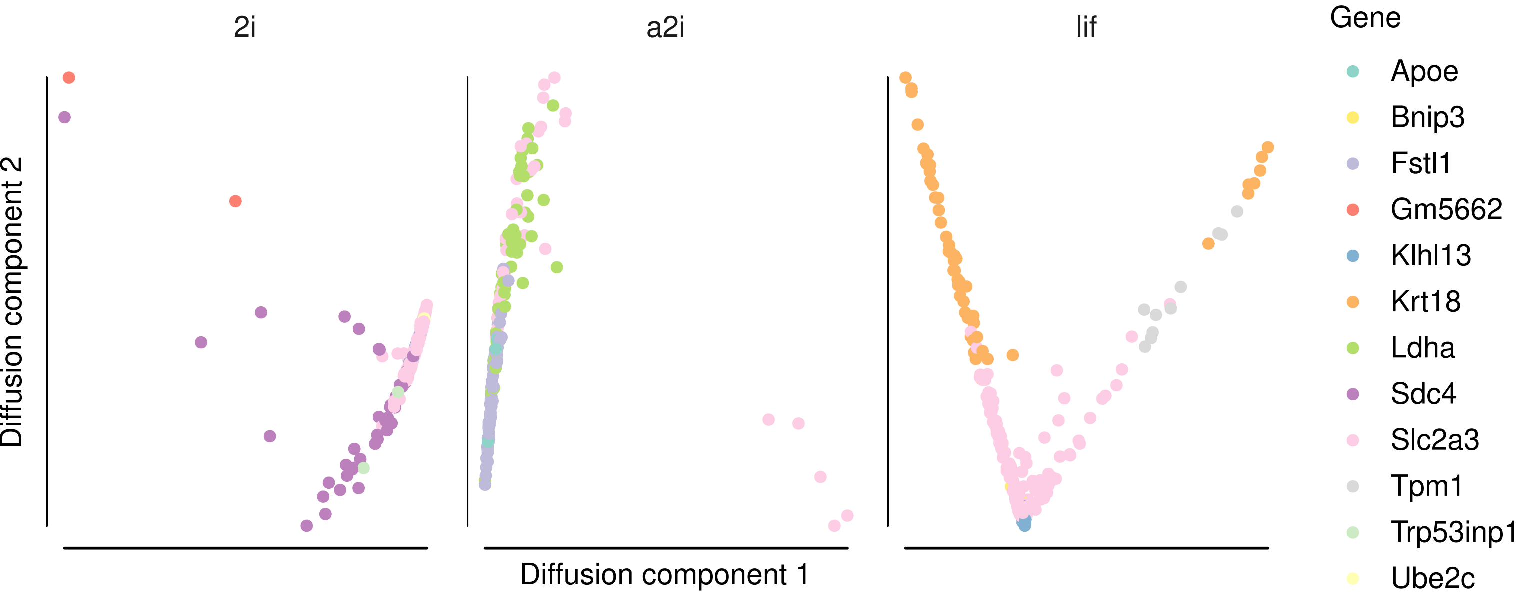
2i
a2i
lif
Diffusion component 2
Diffusion component 1
Gene
Apoe
Bnip3
Fstl1
Gm5662
Klhl13
Krt18
Ldha
Sdc4
Slc2a3
Tpm1
Trp53inp1
Ube2c
