# Supplementary material for: Automatic identification of relevant genes from low-dimensional embeddings of single-cell RNA-seq data
Source: Bioinformatics. 2020 Mar 24;36(15):4291–5. doi: 10.1093/bioinformatics/btaa198 (PMC7520047; doi:10.1093/bioinformatics/btaa198)
Supplement: btaa198_Supplementary_Data [file btaa198_supplementary_data.zip › btaa198-suppl_data/supp-fig6.pdf]

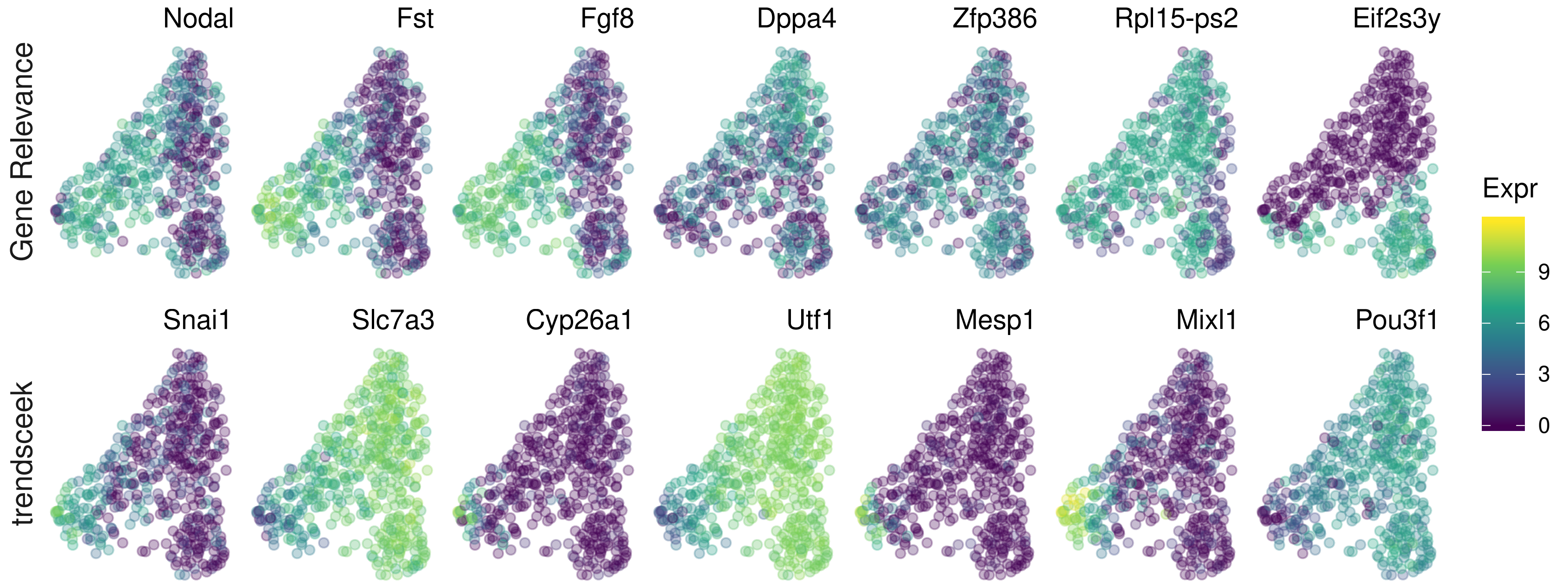
Nodal
Fst
Fgf8
Dppa4
Zfp386
Rpl15-ps2
Eif2s3y
Gene Relevance
Snai1
Slc7a3
Cyp26a1
Utf1
Mesp1
Mixl1
Pou3f1
trendsceek
Expr
9
6
3
0
