# Supplementary material for: Automatic identification of relevant genes from low-dimensional embeddings of single-cell RNA-seq data
Source: Bioinformatics. 2020 Mar 24;36(15):4291–5. doi: 10.1093/bioinformatics/btaa198 (PMC7520047; doi:10.1093/bioinformatics/btaa198)
Supplement: btaa198_Supplementary_Data [file btaa198_supplementary_data.zip › btaa198-suppl_data/supp-fig7.pdf]

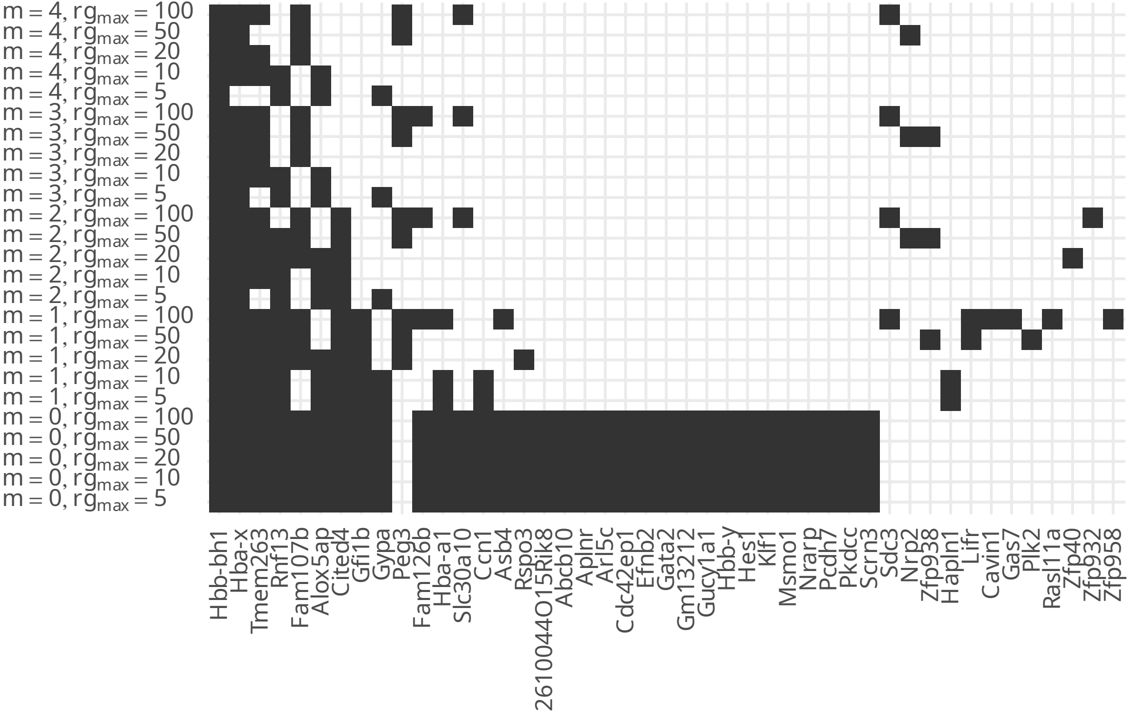
m = 4, rg_{max} = 100
m = 4, rg_{max} = 50
m = 4, rg_{max} = 20
m = 4, rg_{max} = 10
m = 4, rg_{max} = 5
m = 3, rg_{max} = 100
m = 3, rg_{max} = 50
m = 3, rg_{max} = 20
m = 3, rg_{max} = 10
m = 3, rg_{max} = 5
m = 2, rg_{max} = 100
m = 2, rg_{max} = 50
m = 2, rg_{max} = 20
m = 2, rg_{max} = 10
m = 2, rg_{max} = 5
m = 1, rg_{max} = 100
m = 1, rg_{max} = 50
m = 1, rg_{max} = 20
m = 1, rg_{max} = 10
m = 1, rg_{max} = 5
m = 0, rg_{max} = 100
m = 0, rg_{max} = 50
m = 0, rg_{max} = 20
m = 0, rg_{max} = 10
m = 0, rg_{max} = 5
Hbb-bh1
Hba-x
Tmem263
Rnf13
Fam107b
Alox5ap
Cited4
Gfi1b
Gypa
Peg3
Fam126b
Hba-a1
Slc30a10
Ccn1
Asb4
Rspo3
2610044O15Rik8
Abcb10
Aplnr
Arl5c
Cdc42ep1
Efnb2
Gata2
Gm13212
Gucy1a1
Hbb-y
Hes1
Klf1
Msmo1
Nrarp
Pcdh7
Pkdcc
Scrn3
Sdc3
Nrp2
Zfp938
Hapln1
Lifr
Cavin1
Gas7
Plk2
Rasl11a
Zfp40
Zfp932
Zfp958
