# Supplementary figures and images for: Automatic identification of relevant genes from low-dimensional embeddings of single-cell RNA-seq data
Source: Bioinformatics. 2020 Mar 24;36(15):4291–5. doi: 10.1093/bioinformatics/btaa198 (PMC7520047; doi:10.1093/bioinformatics/btaa198)

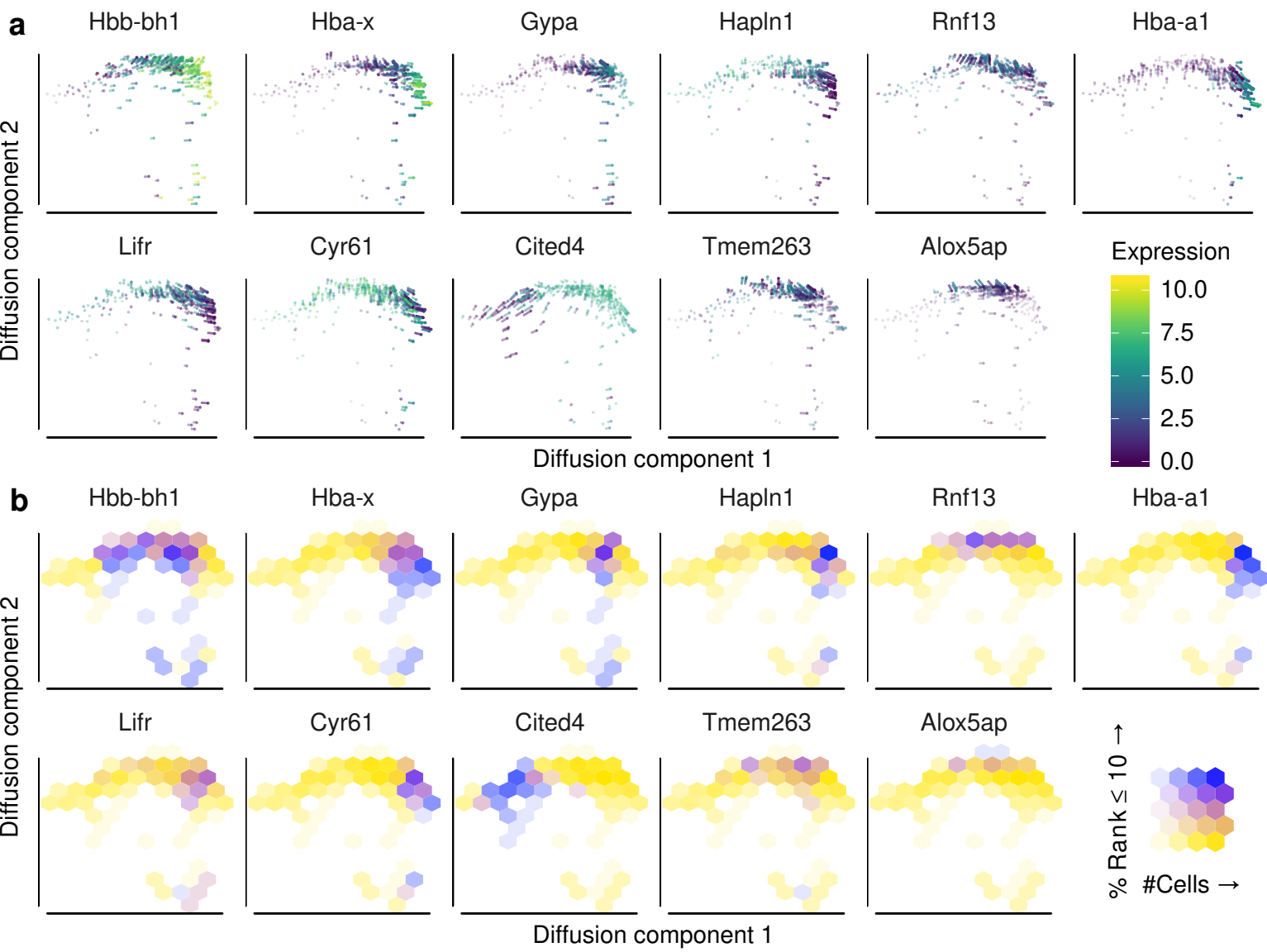

Supplement: btaa198_Supplementary_Data [file btaa198_supplementary_data.zip › btaa198-suppl_data/supp-fig1.pdf]

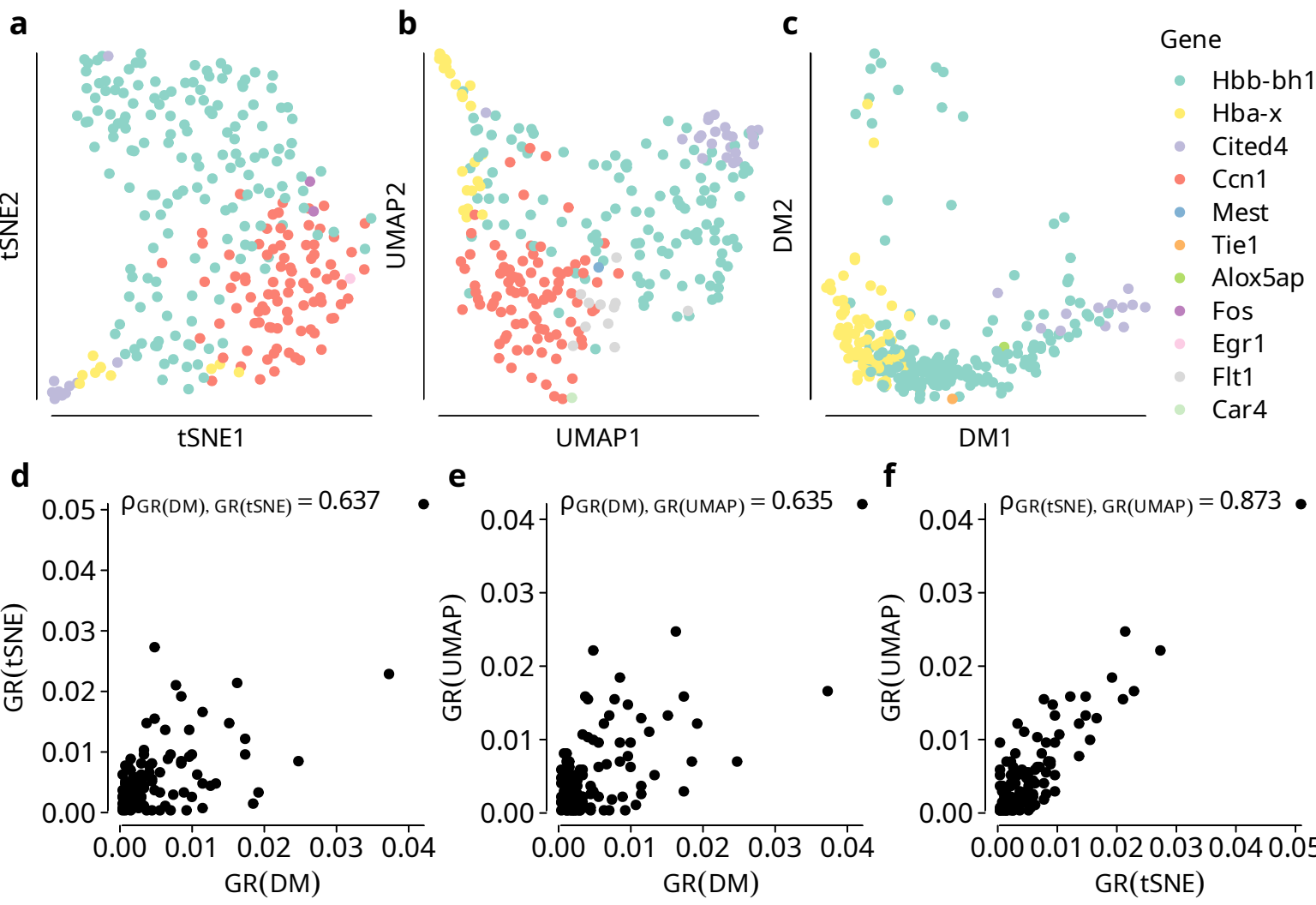

Supplement: btaa198_Supplementary_Data [file btaa198_supplementary_data.zip › btaa198-suppl_data/supp-fig2.pdf]

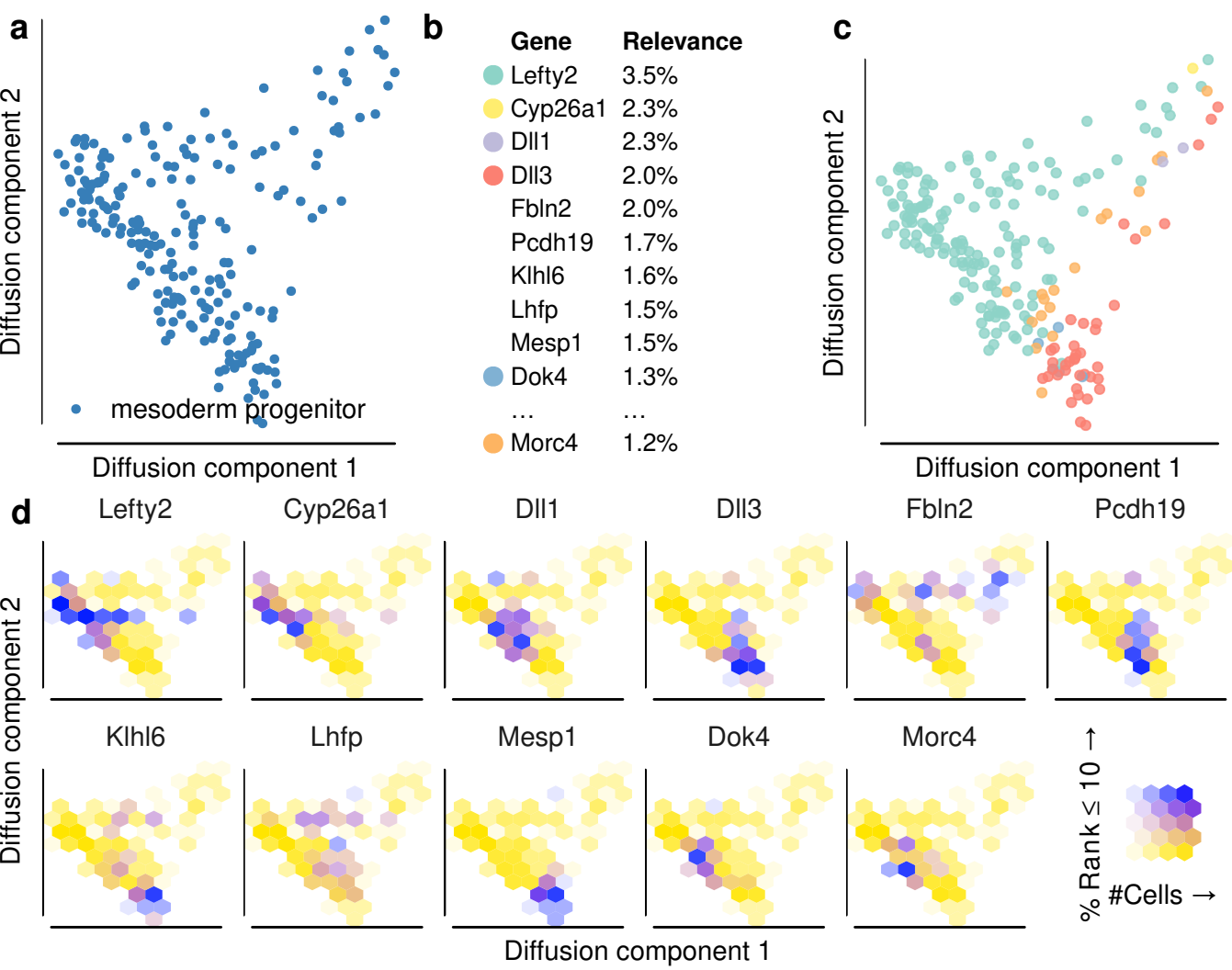

Supplement: btaa198_Supplementary_Data [file btaa198_supplementary_data.zip › btaa198-suppl_data/supp-fig4.pdf]

PCA loading

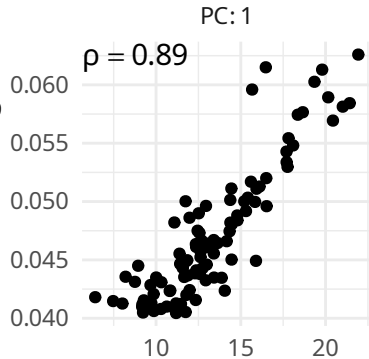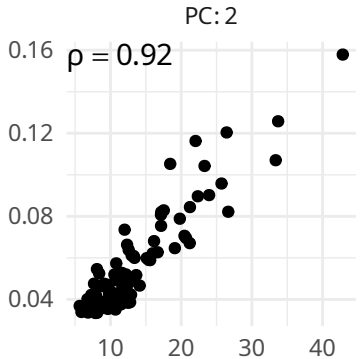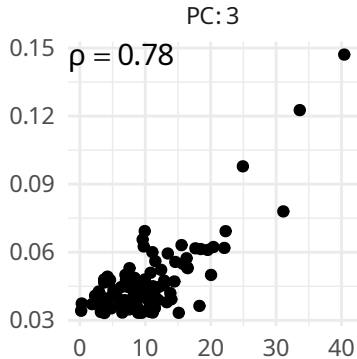

global gene relevance

Supplement: btaa198_Supplementary_Data [file btaa198_supplementary_data.zip › btaa198-suppl_data/supp-fig8.pdf]
